# Toxic Shock Syndrome Due to Streptococcus pyogenes: A Case Series

**DOI:** 10.7759/cureus.91342

**Published:** 2025-08-31

**Authors:** Tiago Tavares, Jerónimo Domingos, Valériya Zaruba, Carla Nobre, Manuel Sousa

**Affiliations:** 1 General Surgery, Unidade Local de Saúde da Arrábida (ULSA), Setúbal, PRT; 2 Intensive Care Unit, Unidade Local de Saúde da Arrábida (ULSA), Setúbal, PRT

**Keywords:** clindamycin, intensive care unit, necrotizing fasciitis, streptococcus pyogenes, superantigen, toxic shock

## Abstract

Streptococcal toxic shock syndrome (STSS) is an acute and life-threatening illness with a high mortality rate. It is the result of an infection by group A *Streptococcus*, which can produce exotoxins with superantigen activity. Herein, we report three patient cases who presented to the emergency department with skin lesions and were in a state of shock. In each case, the clinical presentation indicated a diagnosis of toxic shock syndrome, which was posteriorly confirmed when *Streptococcus pyogenes* was isolated in their blood cultures. Only two of the cases were treated with clindamycin and an antibiotic in the penicillin group; these patients later presented with clinical improvement and had a full recovery. The cases presented demonstrate the rapid evolution of this syndrome, from a skin lesion to fulminant multi-organ failure. Hence, we highlight the need for alertness to this syndrome because once the patient has reached multi-organ failure, halting the disease progression may not be possible without proper treatment and assistance from a multidisciplinary team.

## Introduction

Infections due to streptococcal microorganisms can have diverse presentations, from simple pharyngitis to more severe forms, such as bacteraemia, endocarditis, or deep infections in subcutaneous tissues, such as necrotising fasciitis [1-4]. Toxic shock syndrome occurs as a complication of invasive infection by Group A *Streptococci* (*Streptococcus pyogenes*) [4,5]. The illness occurs in patients of all ages and immunocompetent individuals as an immunological response to the toxins produced by this organism: NADase [4]. Treatment of these patients typically demands a stay in an intensive care unit (ICU) for multi-organ support and infection source control; in patients with necrotising fasciitis, surgery may be required.

## Case presentation

Case 1

A 72-year-old Caucasian woman who was previously autonomous had a relevant personal history of non-insulin-dependent diabetes mellitus and ischaemic cardiopathic disease, which was complicated by cardiac failure. She presented to the emergency department (ED) with high fever (40.5ºC), vomiting, diarrhoea, and marked oedema of the leg, of unknown time of evolution. The physical exam showed a Glasgow coma scale (GCS) of 15, blood pressure of 126/64 mmHg, heart rate (HR) of 103 bpm, and an enlarged left leg with blush. The leg was painful and warm to the touch. 

Laboratory tests (Table 1) on admission showed elevated plasma C-reactive protein (CRP 50 mg/dL), a leucocyte count of 13.8 10^3/uL, an estimated glomerular filtration rate of 28 mL/min/1.73 m^2^, and elevated levels of creatinine kinase (5,350 U/L). An arterial blood gas analysis (BGA) demonstrated hyperlacticaemia (lactates of 3 mmol/L) and type 1 respiratory failure (Fi 21%, pCO_2_ 33 mmHg, and pO_2_ 77.6 mmHg). 

**Table 1:** Laboratory values at admission of case 1

Parameters	Patient values	Reference range
Haemoglobin	14.4 g/dL	11.5–15 g/dL
White cells	13.8 10^3^/uL	4.5–11.4 10^^3^/uL
C-reactive protein	50 mg/dL	<0.5 mg/dL
D-dimer	10,396 ng/mL	<500 ng/mL
Urea	65 mg/dL	19–44 mg/dL
Creatinine	2.16 mg/dL	0.7–1.25 mg/dL
Glomerular filtration rate	28 mL/min	>90 ml/min
Creatinine kinase	5,350 U/L	30–200 U/L
Lactates	3 mmol/L	0.5–2.0 mmol/L
pCO_2_	33 mmHg	32–45 mmHg
pO_2_	77.6 mmHg	80–105 mmHg

**Figure 1:**
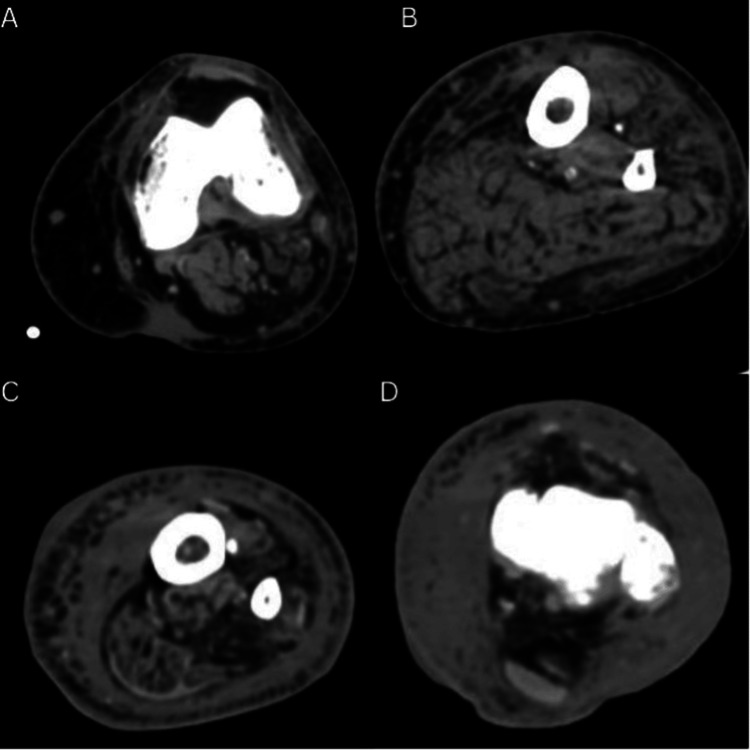
CT scan of the left leg at different levels (A – D) Severe oedema at the subcutaneous level involved all of the leg (at the knee level (A); upper third of the leg (B); lower third of the leg (C)). The muscular plane is shown without any alterations. The ankle region (D) features more prominent oedema.

A CT scan of the thorax disproved the hypothesis of pulmonary embolism, whereas a CT of the leg (Fig. 1) demonstrated the presence of a substantial amount of oedema in the entire length of the leg in the subcutaneous plane. 

During the diagnostic exams, the patient presented with an aggravation of her clinical state, hypotension, and tachycardia with no response to fluid resuscitation, as well as worsening respiratory failure (arterial BGA with a high flow mask (8 L/min with 35% of FiO_2_): pH 7.32, pCO_2_ 41 mmHg, pO_2_ 63 mmHg, HCO_3_ 21.1 mmol/L, and Lac of 5.4). 

The patient was diagnosed with septic shock of cutaneous origin in the leg and was transferred to the ICU. Staff initiated fluid therapy, oxygen therapy, and empirical antibiotic therapy with ceftazidime and clindamycin (due to the high suspicion of toxic shock syndrome). 

The cellulitis in the lower leg evolved to blisters and cutaneous necrosis of the lower third of the leg. The patient underwent surgical debridement of the necrotic areas, and synovial fluid was collected for bacteriological study. On the fourth day, the patient began hyperbaric oxygen therapy and underwent three consecutive sessions. Synovial fluid testing and blood cultures revealed *S. pyogenes*.

The patient's altered mental status persisted. This was initially assumed to be due to the sepsis; however, a lumbar puncture revealed findings consistent with bacterial meningitis. Consequently, the medical team switched to ceftriaxone and clindamycin and initiated dexamethasone therapy. 

The patient's clinical state gradually improved; she was discharged from the ICU on the ninth day of her stay and from the hospital on the 16th day of her stay. 

Case 2

A 37-year-old Caucasian man who was previously autonomous and had no known prior disease presented to the ED due to enlargement of the right lower limb associated with impotence, a high-grade fever, and watery dejections, with approximately three days of evolution. The patient had a GCS of 15; he was conscious and oriented but agitated and complaining of high pain intensity in the right leg. The physical exam revealed an exuberated oedema and stiffness throughout the lower limb and a positive Homan's sign. 

**Figure 2:**
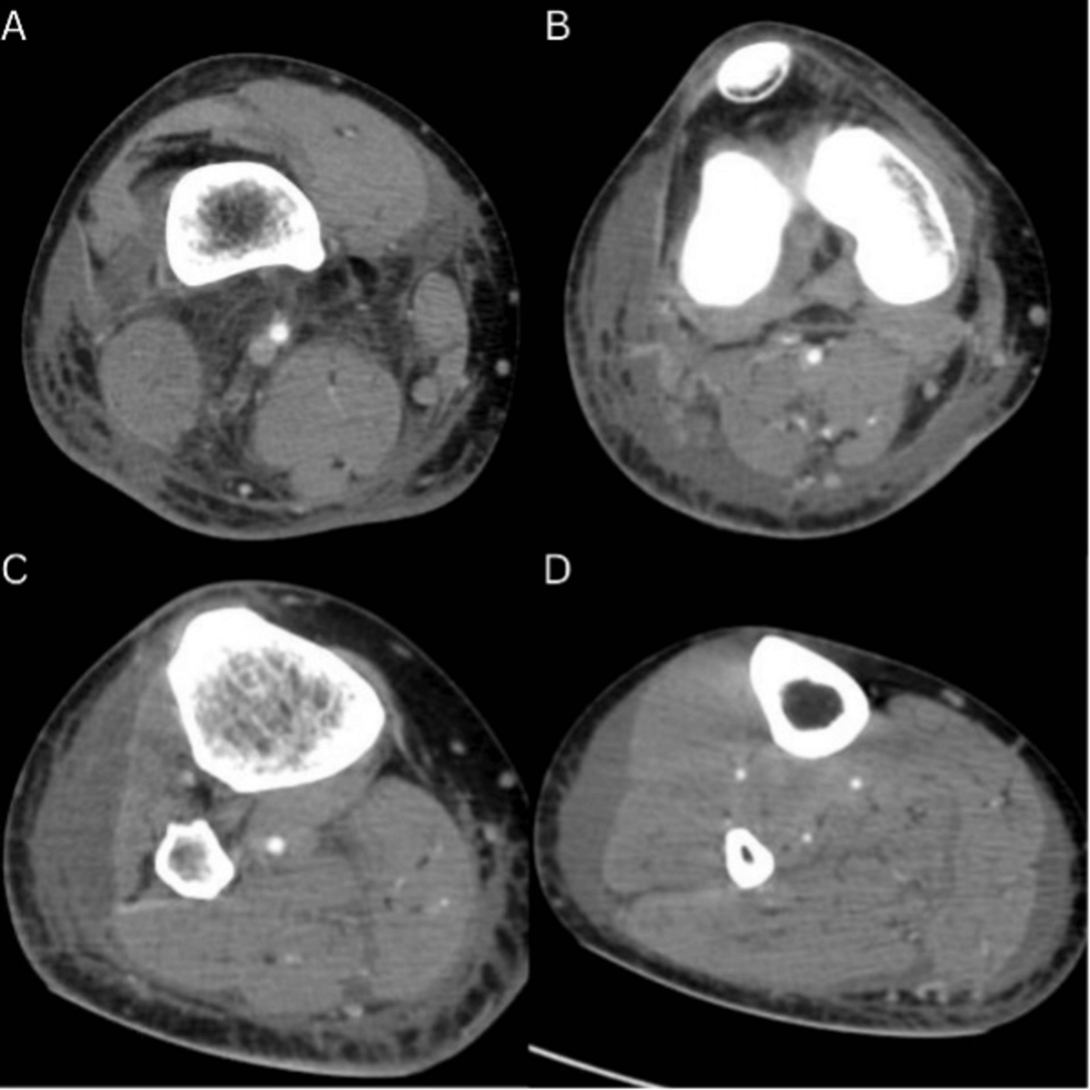
CT scan of the right leg at different levels (A-D) A) In the thigh, one can observe extensive oedema both at epifascial and subfascial levels. B-C) On an intermuscular plane of the hamstring muscles, the oedema can be observed in a concentric fashion as collections in front of the anterior compartment of the leg muscles; the oedema measures approximately 86 mm axially and is approximately 20 mm thick. D) Collection superficial to the distal third of the gastrocnemius with 51 mm of longitudinal width; the collection is parallel to the cutaneous surface.

A CT scan (Fig. 2) showed multiple adenomegalies in the inguinal region, marked oedema in the thigh region with more prevalence in the intermuscular plane, an 86 mm x 20 mm collection (width and depth, respectively) in the anterior compartment of the leg, and a 51 mm x 24 mm collection superficial to the distal third of the gastrocnemius. 

Blood analysis (Table 2) indicated leukopenia (leu 1.7 10^3^/uL) and a high CRP (39.26 mg/dL), acute renal failure, and rhabdomyolysis (CK 852 U/L, myoglobin >1,200 ng/mL). Arterial BGA suggested type I respiratory failure and a lactate of 5.2 mmol/L. 

**Table 2:** Laboratory values at admission of case 2

Parameters	Patient values	Reference range
Haemoglobin	14.2 g/dL	11.5–15 g/dL
White cells	1.7 10^^3^/uL	4.5–11.4 10^3^/uL
C-reactive protein	39.26 mg/dL	<0.5 mg/dL
D-dimer	5,200.0 ng/mL	<500 ng/mL
Urea	134 mg/dL	19–44 mg/dL
Creatinine	6.51 mg/dL	0.7–1.25 mg/dL
Glomerular filtration rate	11 mL/min	>90 ml/min
Creatinine kinase	852 U/L	30–200 U/L
Myoglobin	>1,200 ng/mL	0.3–154.9 ng/mL
Troponin I	6,683.4 pg/mL	28.9–39.2 pg/mL
Lactates	5.2 nmol/L	0.5–2.0 nmol/L
pH	7.4	7.35–7.45
pCO2	35.0 mmHg	77.6 mmHg
pO2	58.4 mmHg	80–105 mmHg
HCO3^-^	21.9 mmol/L	21.8–26.2 mmol/L

While awaiting the exam results, the patient entered into a state of shock and was diagnosed with septic shock of cutaneous origin in the leg (necrotising fasciitis). He was given broad-spectrum antibiotics with piperacillin and tazobactam, underwent a fluid challenge, and was taken to the operating room (OR) for an emergency fasciotomy. 

In the OR, his state of shock was refractory to the previous measures; vasopressor therapy with norepinephrine (maximum dose of 2.2 ug/Kg/min) was initiated. Vancomycin and clindamycin were added to the antibiotic therapy, and the patient was transferred to the ICU for multi-organ support. 

On admission to the ICU, the patient progressed into multi-organ dysfunction: cardiac failure (a bedside echocardiogram was performed, which revealed a severe heart dysfunction and reduced cardiac output (CO 3.8 L/min)), renal failure, acute respiratory distress syndrome, and circulatory dysfunction (refractory hypotension to vasopressor therapy). The patient was transferred to another hospital, where extracorporeal membrane oxygenation was initiated. 

Microbiological results of the haemocultures and the culture of the pus collected from the leg abscesses displayed growth of *S. pyogenes*. After 21 days in ECMO, therapy and organ support were gradually discontinued. The patient later began a rehabilitation programme and follow-up with plastic and reconstructive surgery; he achieved a full recovery after approximately three months of hospitalisation. 

Case 3

A 75-year-old Caucasian woman with a known medical history of non-insulin-dependent diabetes mellitus and ischaemic cardiopathic disease, which was complicated by cardiac failure, atrial fibrillation, obesity, and mild dementia since January of 2023, presented to the ED due to a three-day evolution of altered mental status and severe diarrhoea, which was concomitant with vomiting and a de novo erythematous rash on the lower limb that began on the day of the ED visit. 

The patient also had a high fever of 40ºC, was haemodynamically unstable (MAP of 40 mmHg and bradycardic), and was experiencing polypnoea and a decrease in consciousness level (GCS 8). Her extremities were cold to the touch, and the right lower limb presented with signs of cellulitis and tight skin. Her arterial BGA of 10 L/min of O_2_ indicated severe metabolic acidosis; lactates were 6.0 mmol/L. A blood analysis (Table 3) showed leucocytosis and an increase in CRP, renal dysfunction, hepatic dysfunction, coagulopathy, and thrombocytopaenia.

**Table 3:** Laboratory values at admission of case 3

Parameters	Patient values	Reference range
Haemoglobin	10.9 g/dL	11.5–15 g/dL
White cells	12.9 10^^3^/uL	4.5–11.4 10^3^/uL
C-reactive protein	26.32 mg/dL	<0.5 mg/dL
INR	4.3	0.8–1.2
Platelet count	115,000 10^^3^/uL	150,000–350,000 10^3^/uL
Urea	59 mg/dL	19–44 mg/dL
Creatinine	1.57 mg/dL	0.7–1.25 mg/dL
Glomerular filtration rate	46 ml/min	>90 ml/min
Creatinine kinase	729 U/L	30–200 U/L
Troponin I	315.8 pg/mL	28.9–39.2 pg/mL
Lactates	6.0 nmol/L	0.5–2.0 nmol/L
ASAT	102 U/L	5–34 U/L
ALAT	33 U/L	<55 U/L
LDH	334 U/L	125–230 U/L
Total bilirubin	8.26 mg/dL	<1.2 mg/dL
Direct bilirubin	6.37 mg/dL	<0.5 mg/dL
pH	7.321	7.35–7.45
pCO2	27.8 mmHg	77.6 mmHg
pO2	77.6 mmHg	80–105 mmHg
HCO3^-^	16.2 mmol/L	21.8–26.2 mmol/L

**Figure 3:**
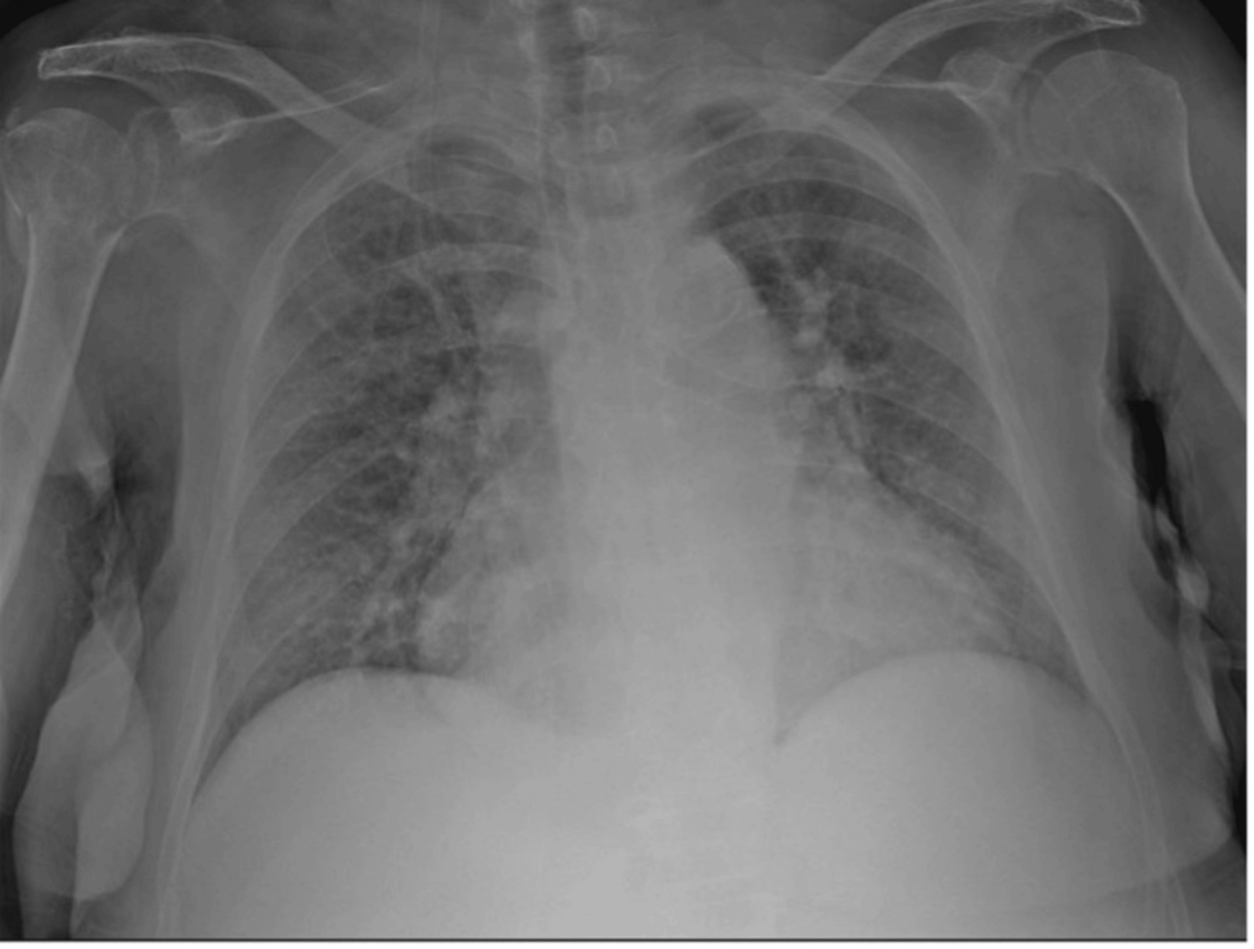
Chest X-ray with bilateral opacities

**Figure 4:**
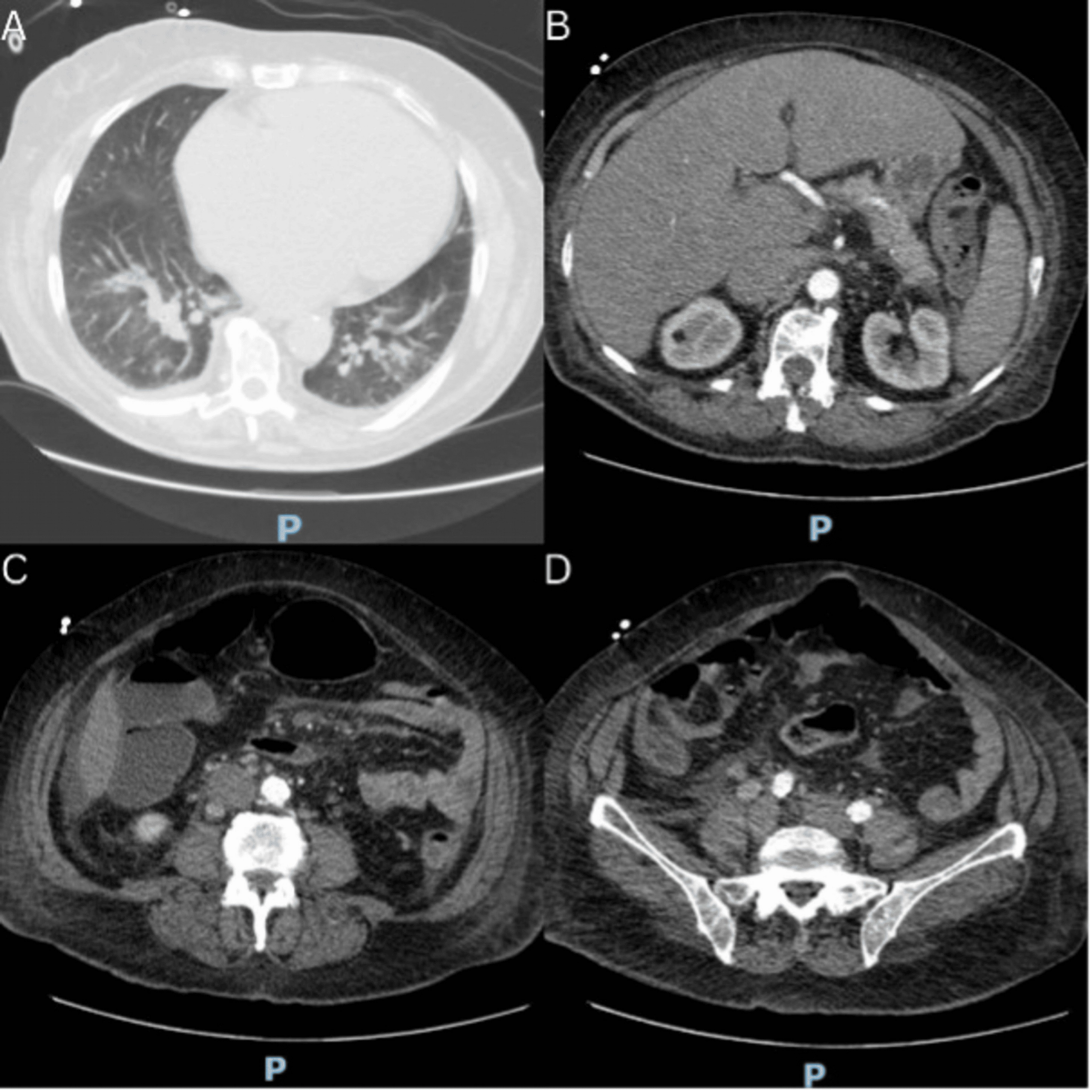
Full-body CT scan at different levels (A–D) A) Severe cardiomegaly and mild pleural effusion on the right (with 2 mm of thickness). B) Hepatomegaly; the liver measures 20 cm at the midclavicular line and features generalised reduction of its density. Dilatation of the retrohepatic inferior vena cava and of the suprahepatic veins reveals reflux of the contrast to the suprahepatic veins during the arterial phase. Mild splenomegaly (spleen measuring at 13.2 cm). C–D) Mild dilatation of the inferior vena cava. Multiple latero-aortic adenopathy is seen (the largest with a 1.3 cm diameter) in addition to some adenopathy in the common iliac chain (the largest is 1.1 cm) and in the inguinal region (the largest is 1.7 cm).

A chest X-ray (Fig. 3) demonstrated bilateral opacities in all lobes of the lungs. A full-body CT scan (Fig. 4) showed signs suggestive of congestive heart failure. A transthoracic echocardiogram showed mild aortic insufficiency and an inferior vena cava dilatated with reduced inspiratory collapse (DAP of 15 mmHg). The ejection fraction was 35%. 

Septic shock was assumed to be the most likely diagnosis, with the probable origin being the cellulitis of the lower limb; hence, piperacillin and tazobactam were initiated. 

Similar to the previous case, an unfavourable devolvement of metabolic acidosis (increase of the hyperlacticaemia) and respiratory exhaustion required that the patient be placed under invasive mechanical ventilation; she was later transferred to the ICU, where cardiovascular support was initiated with norepinephrine (maximum dose of 0.9 ug/Kg/min), 1,500 ml of lactate ringer, and 250 mg/50 ml (3 ml/h) of dobutamine, with a posterior increase of MAP to 70 mmHg. 

A few hours after being admitted to the ICU, the patient developed fulminant multi-organ failure and eventually suffered cardiorespiratory arrest. The patient was declared dead less than 24 hours after being admitted to the ED. 

In the three aforementioned cases, the final diagnosis was toxic shock syndrome that began with a skin lesion that led to cellulitis due to an infection by *S. pyogenes*. 

## Discussion

Streptococcal toxic shock syndrome (STSS) is a serious and severe complication of an invasive infection by a gram-positive species that can cause disease through toxin production [4]. Most cases are the result of an infection by a group A *Streptococcus* (*S. pyogenes*) microorganism [5,6]. STSS has a sudden onset and leads to a rapid multi-systemic organ dysfunction, which results in shock and multi-organ failure early in its clinical course [5].

Worldwide, the incidence of STSS is 3.5 cases per 100,000 people per year, with a higher incidence found at the extremes of ages, in patients with chronic illnesses (such as diabetes or other diseases that cause an immunocompromised state), and in association with intake of nonsteroidal anti-inflammatory drugs [4-7]. The most recent studies have detailed the mortality rate to be between 23% and 44%; however, this rate increases to 50% when associated with necrotising fasciitis [4-6].

Streptococci are among the few bacteria known to produce exotoxins with superantigen capacity; these proteins can induce a high-intensity activation of nonspecific T cells, which they achieve through bypassing conventional mechanisms of MHC-limited antigen cell activation. This initiates a downstream stimulation of other inflammatory cell types, thus causing a release of cytokines and chemokines [2-5,8,9]. This flood of pro-inflammatory cytokines and other mediators leads to a generalised inflammatory response that explains the capillary leak, arterial hypotension, and multi-organ dysfunction seen in patients with STSS [2,9]. 

One study's findings demonstrate that the antigen activation of the immune system increases its output by over 250 times (normally, <0.1% of lymphocytes are active through a single antigen-triggered T cell activation) [4]. 

Looking at the pathogenesis of STSS, one can come to understand the symptoms that arise from this sort of infection, remembering that the symptoms arise mainly as a body response to a hyper-stimulation of the immune cells that cause them to secrete pro-inflammatory components. 

It has been described in different case series that the clinical presentation of STSS can be divided into three main phases [1,3,4]. The first stage involves nonspecific symptoms, which are characterised by an acute influenza-like illness (high fever of ~40ºC, headaches, chills, and myalgia) and gastrointestinal involvement, such as watery diarrhoea, nausea, and vomiting. Patients with a defined entry portal (injury from a blunt trauma, muscle strain, haematoma, or skin ulcer, etc.) may present with visible signs of inflammation surrounding the entry area. In patients who do not have a defined entry portal, the clinical signs of a deep infection may become evident in a later stage of the illness [2-5,9,10]. 

A pathognomonic symptom of a deep infection associated with STSS is the patient's disproportionate muscle pain to the clinical findings; the pain can be not only uniquely localised to the limb but can also be present as thoracic or abdominal pain [4].

This array of nonspecific signs and symptoms, as well as the rarity of STSS cases, may lead to a premature misdiagnosis; the most common differentials are deep vein thrombosis, limb ischaemia, gastroenteritis, or peritonitis [4,6,11].

Approximately 24-48 hours after the first phase begins, the second phase occurs, in which systemic manifestations emerge, including a severe state of hypotension, tachycardia, tachypnoea, and high fever [4].

The third phase occurs 24-48 hours after the second phase. The disease advances almost unstoppably, leading to circulatory shock and associated multi-organ failure (haematological, cardiac, pulmonary, neurologic, renal, etc.). Despite directed, intensive treatment and organ substitution techniques, most patients succumb to death one or two days after being hospitalised [1-2,4 -5,8-9].

Given the rapid and severe clinical course, a timely and accurate diagnosis becomes crucial. In 2011, the CDC reviewed the 1997 case definition of STSS to create the current diagnostic criteria for the illness. However, the diagnosis is often strenuous since this syndrome does not present specific symptoms that enable differentiation from another sort of septic shock [4,12].

The CDC criteria divide patients into two categories: probable and confirmed cases. In both situations, the patient has positive clinical criteria that are based on the presence of signs and symptoms of shock, organ dysfunction (renal, haematological, hepatic, cardiac, and respiratory), and skin lesions (generalised erythematous macular rash and soft tissue necrosis that may include necrotising fasciitis, myositis, or gangrene). What separates one category from the other is the second diagnostic criterion: the laboratory criteria, which are considered positive when there is isolation of group A *Streptococcus* organisms on a sterile site (blood, cerebrospinal, synovial, pleural, or pericardial fluid). If a patient has positive clinical criteria (gastrointestinal symptoms, such as diarrhoea, vomiting, or abdominal pain; severe disproportionate pain; signs of skin and soft tissue infection; a generalised erythematous macular rash, which later begins to desquamate; or multi-organ failure of two or more of the aforementioned systems) and no laboratory criteria (no isolation of GAS), then it is considered a probable case. When there is an isolation of GAS from a sterile site, then STSS is confirmed [2,4].

Clinicians should bear in mind the possibility of STSS when dealing with patients who develop a rapid onset of shock, associated with a skin rash, with or without associated necrotising fasciitis [2,3,12].

As mentioned previously, most diagnoses are made in a later stage of the disease when there is already advanced systemic dysfunction, represented by multi-organ failure. At this point, ICU admission is common, and supportive techniques must be initiated [4]. The same basic therapeutic strategy for septic shock should be applied: active fluid resuscitation and early use of vasopressors and inotropes. Most patients require renal replacement techniques and mechanical invasive ventilation. Empirical therapy with broad-spectrum antimicrobial agents should begin immediately after cultures have been taken [4,11-12].

A rapid diagnosis is of critical importance, as is rapid treatment to halt the progression of the disease and to reduce to a minimum both the morbidity and mortality of STSS. To do this, one must eradicate the source of the toxin production, which can occur through two major medications: a bacteriostatic agent and a bactericidal agent [2-4,7,11-12]. Although penicillin has been used over the past seven decades, GAS remains sensitive to β-lactam agents, such as penicillin G, which is a bactericidal agent, and in high parenteral doses is a first-line therapy for infections due to GAS [4,11-12]. Findings in recent studies have proven that, particularly in Europe and Asia, GAS has gained resistance to macrolides and fluoroquinolones [2-11].

Clindamycin should be added to the STSS therapy for its bacteriostatic effect. This antibiotic has the capacity to inhibit the synthesis of the 50S subunit of the bacterial ribosome, which prevents group A *Streptococcus* from producing exotoxins that cause STSS. The addition of clindamycin exponentially increases the survival rate of patients affected by STSS [4,8,11].

Surgery is an essential step in the treatment of patients with severe soft tissue infection, especially in patients with necrotising fasciitis or gangrene, because the antibiotic penetration into the infected tissues is reduced, if not absent. Consequently, only surgical debridement of infected and necrotic tissues guarantees the entire removal of agents capable of producing super-antigens. In other words, surgery is a proper source control [1,3,4,12].

Each patient in this study presented to the ED with symptoms that were compatible with lower limb cellulitis, which was preceded by two to three days of high fever, nausea, and vomiting. All the patients developed, within 24-48 hours’ time, multi-organ failure (renal, respiratory, cardiovascular, haematological, and/or hepatic). Blood cultures were collected before antibiotic therapy began; all cultures revealed isolated *S. pyogenes* agents. The patients were admitted to the ICU for monitoring and organ support therapies. In general, all were medicated with a β-lactam agent; however, only two were concomitantly medicated with clindamycin. When necessary, surgical debridement of the infected tissues was performed. Despite the efforts involved, one patient developed unfavourably, although the other two patients evolved positively, with complete functional recovery.

## Conclusions

STSS is an acute and life-threatening illness with a high mortality rate that is the result of an infection by group A *Streptococcus* agents, which are capable of producing exotoxins with superantigen activity. Although STSS is a rare disease, the severity associated with this clinical syndrome lends it an emergent characteristic. The survival of these patients depends on rapid diagnosis and appropriate, timely therapy to combat the severity and swift onset of shock and multi-organ failure. The multidisciplinary approach between the specialties of intensive medicine and general surgery is central to the treatment of the patient, which is verified by the clinical cases presented.

In this study, all patients, regardless of sex, age, or comorbidities, first presented with generalised viral symptoms, which subsequently progressed to cellulitis of the lower limb. Once this stage was reached, the disease course was remarkably similar across cases, ultimately leading to multi-organ failure.

A consistent finding, also widely reported in the literature, was the association between clindamycin use and improved prognosis. Prompt surgical management, particularly early debridement of infected tissue, also appeared to play a decisive role in patient recovery.

By contrast, one patient had an unfavourable outcome. This was likely due to a delayed diagnosis, since the patient presented in septic shock, together with the absence of clindamycin in the treatment regimen and the use of non-steroidal anti-inflammatory drugs prior to admission.

By sharing these cases, we aim to enhance awareness among healthcare professionals and underscore the importance of vigilance and coordinated management in improving survival and quality of care.
